# Eating attitudes and food intakes of elite adolescent female figure skaters: a cross sectional study

**DOI:** 10.1186/1550-2783-9-53

**Published:** 2012-12-13

**Authors:** Johanna Dwyer, Alanna Eisenberg, Kathy Prelack, Won O Song, Kendrin Sonneville, Paula Ziegler

**Affiliations:** 1 Frances Stern Nutrition Center, Tufts Medical Center, 800 Washington Street, Box #783, Boston, MA 02111, USA; 2 Jean Mayer Human Nutrition Research Center on Aging, Tufts University, 711 Washington Street, Boston, MA 02111, USA; 3 School of Medicine, Tufts University, 136 Harrison Avenue, Boston, MA 02111, USA; 4 Shriners Hospitals for Children, 51 Blossom Street, Boston, MA 02114, USA; 5 Department of Food Science and Human Nutrition, Michigan State University, Room 135, TFSHN Building, East Lansing, MI 48824, USA; 6 Harvard Medical School, 25 Shattuck Street, Boston, MA 02115, USA; 7 Division of Adolescent Medicine, Children’s Hospital Boston, 300 Longwood Avenue, Boston, MA 02115, USA; 8 College of Saint Elizabeth, 2 Convent Road, Morristown, NJ 07960, USA

**Keywords:** Eating attitudes, Female athletes, Disordered eating, EAT scores, Dietary intake, BMI

## Abstract

**Background:**

Elite adolescent female figure skaters compete in an aesthetic-based sport that values thin builds and lithe figures. To conform to the sport’s physical requirements, skaters may alter their eating patterns in unhealthful directions. This study assesses the eating attitudes and dietary intakes of elite adolescent female figure skaters to assess the potential nutritional risks among them.

**Methods:**

Thirty-six elite competitive adolescent female figure skaters (mean age 16 ± 2.5 SD years) completed self-administered three-day records of dietary intake and simultaneous physical activity records during training season. Two months later, they attended a national training camp during which they completed the Eating Attitudes Test (EAT-40), provided fasting blood samples, and had heights and weights measured.

**Results:**

Participants’ mean body mass index (BMI) was 19.8 ± 2.1 SD. Their BMIs were within the normal range, and the majority (70%) did not report a history of recent weight loss. The mean EAT-40 score was normal (19.5 ± 13.5 SD) and below the cut-off score of 30 that indicates clinically significant eating pathology. However, one-quarter of the skaters had EAT-40 scores above 30. The skaters reported a mean energy intake of 1491 ± 471 SD kcal/day (31 ± 10 SD kcal/kg), with 61.6% of calories from carbohydrate, 14.6% from protein, and 23.7% from fat. Their reported dietary intakes were high in carbohydrates but low in total energy, fat, and bone-building nutrients.

**Conclusions:**

Although these highly active young women compete in a sport that prizes leanness, they had appropriate weights. The athletes reported dietary intakes that were far below estimated energy needs and were at moderate risk of disordered eating. Anticipatory guidance is warranted to improve their dietary intakes, particularly of bone-building nutrients.

## Background

Interest and participation in figure skating has grown consistently over the past 15 years. The US Figure Skating Association USFSA; [1] currently boasts over 176,000 members and 750 member clubs nationwide. While many members participate recreationally, a growing number of athletes strive to join the elite rank of skaters that compete nationally. As the popularity and competition of the sport increases, these figure skaters face growing pressure to complete ever more demanding routines that include advanced jumps and complex technical maneuvers [2-5].

Elite figure skaters must combine strength, endurance and artistry in their on-ice performances. Skaters’ routines are judged based on their technical merit and presentation with subjective evaluation of their artistic perfection and aesthetic appeal [2,4]. Small builds, lean figures, and low body weights are valued attributes in female skaters, for both aesthetic and mechanical reasons [3,4,6,7]. Elite skaters must achieve a sleek, graceful bodily appearance while preserving the power, balance and flexibility a competitive athlete requires [2,3,7,8].

On average, elite adolescent skaters devote 33 hours per week to moderate-to-vigorous physical activity – 27 hours per week to on-ice training and an additional 6 hours per week to off-ice dance and strength training [4]. To promote optimal skating performance, the dietary intakes of figure skaters must meet the energy demands of both intense training and adolescent growth and development [9,10]. However, intense pressures to conform to the sport’s aesthetic ideal, coupled with traditional societal pressures regarding female weight and body shape, could cause skaters to alter their eating and exercise patterns in unhealthful directions [11-13].

Adolescent skaters face a dual challenge, trying to control body weight for a lean-build sport while meeting the high energy demands of training. Prior studies with elite skaters have shown evidence of energy restriction and inadequate energy intake, along with possible inadequacies in key bone-building nutrients, such as vitamin D, calcium, magnesium and zinc [5,7,14-18]. Restrictive eating attitudes and inadequate dietary intake by skaters may lead to a variety of short- and long-term consequences, such as altered athletic performance, fatigue, injuries, amenorrhea and eating disorders [7,9,16].

Knowledge on the dietary practices of this group of elite athletes is ongoing. The purpose of this study was to assess (1) the energy, macro- and micronutrient intakes as well as (2) the eating attitudes of a group of elite adolescent female figure skaters to assess the potential nutritional risks among them. The results of this study will identify potential nutrient inadequacies and disordered eating attitudes and behaviors to inform the nutrition education and counseling needs of elite adolescent female skaters.

## Methods

### Participants

Participants were 36 nationally-ranked elite adolescent female figure skaters who had a mean age of 16 ± 2.5 SD years (range 13–22 years) and who attended an elite training camp at the US Olympic Training Center in Colorado Springs, CO between 1998 and 1999. Written informed consent was obtained from all participants and, where necessary, by their legal guardians prior to participation in the study. The Sports Medicine Advisory Board of the US Figure Skating Association and the US Olympic Committee Human Subject Review Board approved this study. The Human Investigation Review Committee at Tufts Medical Center in Boston, MA approved the secondary analysis of the data.

### Height and weight

Participants were weighed and measured (in light clothing and without shoes) in the morning prior to engaging in physical activity. Body weight was measured using a beam balance scale to the nearest 50 g and height was measured with a stadiometer to the nearest 0.5 cm. Body mass index (BMI) was then calculated as the ratio of weight (kg) to height (m) squared (kg/m^2^); BMI-for-age percentiles were calculated for all participants ≤19y using growth charts from the Centers for Disease Control and Prevention CDC; [19].

### Dietary intake

Dietary intake was assessed to determine the chief sources of energy and key micronutrients in skaters’ diets. After participants were provided detailed instructions, three-day food records (2 nonconsecutive weekdays and 1 weekend day) were recorded during a period of active training two months prior to attendance at the training camp. During the first week of training camp, participants met with a study staff member to review their food records and clarify missing or ambiguous data. The skaters then received a brief individualized nutrition education session with recommendations to help them improve their intakes if they exhibited problems. Diet records were then verified, coded, entered and analyzed by a registered dietitian on the study staff using Nutritionist IV (version 4.1, 1997, First Data Bank, Inc., San Bruno, CA). Estimated intakes of calories, macronutrients, and micronutrients were compared to age and gender appropriate normative data from the National Health and Nutrition Examination Survey of 1999–2000 NHANES; [20-23].

### Eating attitudes

The Eating Attitudes Test EAT-40; [24] served as a measure of disordered attitudes and behaviors towards eating and body weight control. The EAT-40 was originally designed as an objective measure of the symptoms of anorexia nervosa [24]. It was later validated as a broad measure of abnormal eating patterns and is now used as a screening tool for undifferentiated eating disorders in high-risk populations [24,25]. Presently, the EAT-40 is considered the most widely used self-report measure of disordered eating [25] and has been used in prior studies with elite skaters [14,17]. The EAT-40 has a high degree of internal reliability with Cronbach’s alphas ranging from 0.79-0.94 [24]; measures greater than 0.7 are acceptable [26].

The EAT-40 is a self-reported 40-item instrument answered on a 6-point Likert-type scale (1 = *never*, 6 = *always*). The instrument is scored by assigning points to each response (3 points for the most “symptomatic” response, 2 points for the next “symptomatic” response, 1 point for the least “symptomatic” response, and no points for “non-symptomatic” responses) and summing scores for all 40 items [24]. EAT-40 scores >30 indicate the presence of clinically significant eating pathology [24,25].

### Physical activity level

Three 24-hour records of physical activity were collected on the same three days participants recorded their dietary intakes to estimate physical activity level during a period of active training. Participants reviewed the activity records with a study staff member during the first week of training camp to clarify missing or ambiguous data, and means were calculated.

### Blood chemistries

A 12-hour fasting blood sample (25 ml) was obtained by venipuncture from each skater on the first morning after arrival at the training camp and analyzed for hematologic indices (serum iron, total iron binding capacity, total iron saturation, serum ferritin, hemoglobin and hematocrit) and serum albumin (Pikes Peak Diagnostic Service, Inc., Colorado Springs, CO).

### Data analysis

All data were analyzed using the SPSS for Windows statistical program (version 7.0, 1997, SPSS, Inc., Cary, NC). Means and standard deviations were calculated for each variable to provide descriptive information on the anthropometrics, nutrient intake, EAT-40 scores and biochemical indices of nutritional status for the skaters.

## Results

Table 1 describes the characteristics of these competitive adolescent female figure skaters. The 36 participants ranged in age from 13–22 years with a mean and median age of 16 years. The group had a mean BMI of 19.8 ± 2.1 SD (median 19.9) with a range from 15.1 – 23.3. All skaters >19y had normal BMIs compared to adult standards. All but one of the skaters ≤19y had a BMI-for-age within the healthy weight range (5^th^ to 85^th^ percentile) using age- and gender-specific CDC growth charts [19]. Based on these charts, 1 skater had a BMI-for-age <5^th^ percentile and would be classified as “underweight,” 7 skaters were between the 5^th^-25^th^ percentile, 13 skaters were between the 25^th^-50^th^ percentile, 9 skaters were between the 50^th^-75^th^ percentile and 2 skaters were between the 75^th^-85^th^ percentile. Of the 33 skaters who reported weight history, the majority (70%) had no recent weight loss and 25% were actively gaining weight. However, 38% of these skaters considered themselves to be overweight and 22% reported being told by others that they were overweight.

**Table 1:** Descriptive characteristics and estimated energy intake and energy expenditure of elite adolescent female figure skaters (n = 36)

	**Mean ± SD**	**Range**
Age (y)	16.0 ± 2.5	13.0 – 22.0
Height (cm)	158.6 ± 5.8	144.8 – 172.7
Weight (kg)	48.5 ± 6.6	30.6 – 59.1
BMI (kg/m^2^)	19.8 ± 2.1	15.1 – 23.3
Energy Intake (EI)	1491 ± 471	566 – 2654
Estimated Energy Requirement (EER)^a^	2695 ± 154	2314 – 2977

^a^ Equations from 2005 Food and Nutrition Board DRIs [27]; PA = Physical Activity Coefficient.

EER (9-18y) = 135.3 – (30.8 x age[y]) + PA x [(10 x weight[kg]) + (934 x height[m])] + 25.

EER (≥ 19y) = 354 – (6.91 x age[y]) + PA x [9.36 x weight[kg]) + (726 x height[m])].

### Dietary intake and energy expenditure

Table 1 also describes skaters’ estimated energy intakes and expenditures. Mean energy intake (EI), estimated from 3-day diet records, was 1491 ± 471 kcal/day (range 566–2654 kcal/day), which provided a mean 31 ± 10 SD kcal/kg. The average Estimated Energy Requirement (EER), calculated from sex, age, weight, height and reported physical activity levels using Dietary Reference Intake equations DRI; [27] was 2695 ± 154 SD kcal/day (range 2314 – 2977 kcal/day). Compared to energy intakes, skaters had a reported energy deficit (EER minus EI) of 1204 ± 531 SD kcal/day (range from −170 – 2263 kcal/day). Skaters’ reported energy intakes were thus considerably lower (44 ± 19%) than their EERs.

Table 2 shows that these skaters reported a mean 61.6% of energy from carbohydrate, 23.7% from fat, and 14.6% from protein. These intakes provided, on average, 1.2 ± 0.4 g/kg body weight protein and 4.8 ± 1.5 g/kg body weight carbohydrate. Skaters reported a mean 23.8% of energy (91 g/day) from sugar alone. Compared to age- and gender-matched normative NHANES 1999–2000 data, the majority of skaters reported low intakes of key micronutrients including calcium, iron, phosphorus, magnesium, zinc and Vitamin B-12. The majority of skaters (67%) did not take micronutrient supplements.

**Table 2:** Mean daily nutrient intakes of elite adolescent female figure skaters (n = 34)

**Nutrients**	**Elite skaters**	**NHANES 1999–2000** (12-19y)	
	**Mean ± SD**	**Mean ± SEM**	**% NHANES**
Energy (kcal)	1491 ± 471	1993 ± 45.7^a^	75%
Protein (g)	55.8 ± 19.5	67 ± 1.2^a^	84%
Carbohydrate (g)	234.8 ± 70.8	277 ± 3^a^	85%
Fat (g)	40.2 ± 21.9	43 ± 1^a^	93%
Saturated Fat (g)	13.8 ± 7.5	24 ± 0.3^b^	58%
Calcium (mg)	763.3 ± 438.1	793 ± 26.5^c^	96%
Iron (mg)	11.6 ± 4.7	13.4 ± 0.4^c^	87%
Phosphorus (mg)	737.4 ± 345.7	1093 ± 27.3^c^	67%
Magnesium (mg)	183.0 ± 86.8	216 ± 5.7^c^	85%
Zinc (mg)	5.5 ± 2.8	9.6 ± 0.29^c^	57%
Vitamin D (mcg)	2.8 ± 2.6	N/A	N/A
Vitamin B12 (mcg)	2.2 ± 1.6	3.4 ± 0.2^d^	65%

^a^ Reference [23].

^b^ Reference [21].

^c^ Reference [20].

^d^ Reference [22].

Table 3 details the percent contribution of each food group to the estimated intake of key nutrients. Grains contributed the most (35%) to overall energy intake, followed by meat (17%), milk (13%) and sugary foods (9%). Sugar came mainly from fruit (25%), followed by added sugar (20%), milk (15%) and sweetened beverages (12%). Milk was the greatest contributor to bone-building nutrients such as calcium (55%), vitamin D (77%), and phosphorus (36%) intake, followed by the grains group. Grains provided the most iron (56%) and magnesium (34%).

**Table 3:** **Percent (%) contribution of food group to nutrient intakes of elite adolescent female figure skaters**^**ab**^

	**Calcium**	**Iron**	**Magnesium**	**Phosphorus**	**Vitamin D**
Milk	55	5	16	36	77
Meat/Egg/ Legume/Nut/Seed	8	18	13	18	3
Grain	19	56	34	29	12
Fruit	4	4	15	4	1
Vegetable	5	10	14	9	1
Fat/Sugar	2	2	4	1	6
Beverage/Water	6	1	4	3	0
Other	2	4	1	0	0

^a^Foods were grouped together by USDA food group definitions. Water group included mineral and tap water. Other group included condiments and spices.

^b^ Contribution (%) = (∑ Amount of nutrient contributed by the particular food group for an individual / ∑ Total amount of nutrient from all foods for an individual) x 100.

### Eating attitudes test (EAT-40) scores

Mean EAT-40 scores for the skaters were 19.5 ± 13.5 SD (range 6 – 62). Eight of the thirty-three skaters (24%) scored above the EAT-40 cut-off score of 30 that suggests a risk of clinically significant eating pathology. Skaters with elevated EAT-40 scores tended to be older and to have higher BMIs than skaters without elevated scores; there were no differences in reported energy intakes between the groups. Questions with the most affirmative responses from skaters involved restrained eating (“[Do not] enjoy trying new rich foods” (85%), “Display self control around food” (55%), and “Aware of the calorie content of foods that I eat” (42%)), preoccupation with weight (“Am terrified of being overweight” (33%), “Am preoccupied with a desire to be thinner”(33%)) and preoccupation with food (“Give too much time and thought to food” (30%)) in rank order. Skaters also endorsed disliking tight fitting clothing, not enjoying meat, and not having regular menstrual periods. Items regarding pathological weight control (“Vomit after I have eaten” and “Take laxatives”) had the lowest rates of endorsements.

### Biochemical measures

Table 4 summarizes the key blood chemistries. All means for iron and hematologic indices (serum iron, total iron binding capacity, total iron saturation, serum ferritin, hemoglobin and hematocrit) were within normal limits. Only 1 skater, who would be classified as underweight based on BMI-for-age, had both a low serum iron and low percent (%) iron saturation, but all other values for this skater were normal. Overall, there was no evidence of iron deficiency or anemia from the group mean biochemical values. All skaters had serum albumin values within the desired ranges for age.

**Table 4:** Blood iron and lipid indices for elite adolescent female figure skaters

	**N**	**Mean ± SD**	**Range of normal values**^**a**^	**% skaters with abnormal values**
Serum iron (mcg/dL)	36	121 ± 41	60–160 (>18y) 50–120 (≤18y)	44% (3% below range)
Total iron binding capacity (mcg/dL)	36	354 ± 46.7	250–460	3%
Total iron saturation (%)	36	34.8 ± 12.7	15–50%	19% (5.5% below range)
Serum ferritin (ng/mL)	33	40.4 ± 30	10–150	3%
Hemoglobin (g/dL)	36	13.9 ± 0.8	12–16 (>18y) 10–15.5 (≤18y)	0%
Hematocrit (%)	36	40.4 ± 2.5	37–47 (>18y) 32–44 (≤18y)	6% (0 below range)
Albumin (g/dL)	36	4.6 ± 0.3	3.5–5 (>18y) 4–5.9 (≤18y)	0%

^a^Mosby’s Diagnostic and Laboratory Test Reference [28].

## Discussion

Among a sample of competitive adolescent female figure skaters, most had appropriate weights-for-heights. One-quarter of the skaters had an EAT-40 score above 30 which is indicative of clinically significant eating pathology. The skaters did report low intakes of energy and bone-building nutrients, but the majority (70%) reported no recent weight loss and all biochemical measures indicative of iron status were within the normal range. Although the mean EAT-40 score did not indicate risk of disordered eating, there were several athletes (24%) who were at high risk and, among the entire sample, the response pattern did suggest that skaters had heightened awareness of eating restraint and potential preoccupation with weight and food. Like other lean-build athletes, these athletes are at elevated risk for disordered eating, caloric restriction, low-nutrient intakes and weight-loss behaviors [5,16-18,29]. Prior studies with similar athletes report that dietary inadequacies and inappropriate behaviors to control weight are common [2,7,8,11-15,30]. Lean-sport athletes, especially females, report greater pressure to maintain a thin, lithe figure and low body weight than athletes in sports with less emphasis on such builds, and they are at risk of developing preoccupation with weight and body shape that may increase the likelihood of adopting extreme weight loss methods and patterns of disordered eating [11,12].

The elite adolescent female skaters in this study were of normal body weight, despite their low reported energy intakes. Only one of the 36 skaters was classified as “underweight” by BMI-for-age and the mean BMI of 19.8 ± 2.1 SD of the group was similar to that reported in prior studies with elite adolescent skaters [5-8,14-16,30]. However, 38% of the skaters who reported weight history considered themselves to be overweight, and 22% reported being told by others they were overweight. Skaters are involved in a lean-build sport and may perceive pressure to alter their appearance, even if they are of healthy weights. Prior research suggests that training staff (coaches, officials, partners) are integral to skaters self-perceptions on body weight and stature [6,29]. Therefore, it is important for training staff and skaters to understand healthful BMI ranges for elite athletes. Nutrition education efforts should focus on helping skaters understand the relationship between weight and health and learn methods to maintain optimal weight-for-height while meeting the physical demands of the sport with nutrition intervention. In addition, training staff should monitor skaters’ BMIs as undesirable BMI changes may be a warning sign of unnecessary energy restriction and weight loss.

The mean dietary intakes of energy, macro- and micronutrients recorded by skaters in this study were similar to intakes previously reported by elite skaters [5,8,15-17], but were lower than average when compared to normative age- and gender-matched intake data from NHANES 1999–2000 [20-23]. Based on reported EI and EER, the skaters had a reported energy deficit of 1204 ± 531 SD kcal/day. However, skaters’ body weights and BMIs were within normal range and the majority reported no downward trends in weight over time. Therefore, it is likely the dietary intake data were subject to either underreporting of food intake or overestimation of physical activity level. The degree of underreporting in this study (44%) was very high when skaters’ reported EIs were compared to their EERs; the usual degree of underreporting is estimated between 10-20% [31]. Underreporting on food intakes is common, particularly among adolescents and athletes, and the process of recording food intake may cause individuals to alter their dietary patterns [31,32]. The large discrepancy reported in this group may be due to the inevitable limitations involved in having adolescents keep unsupervised food records or, perhaps, to skaters’ attempts to record intakes they perceive their coaches and peers will deem desirable.

The percent contribution of each macronutrient to total intake was similar to recommendations for athletes of 55-60% carbohydrate, 12-15% protein and 20-35% fat [10,33] and similar to results from previous skater studies [15,30]. The main contributors to energy and bone-building nutrients, similar to other studies [14,30], were the grain, meat, milk and sugary food groups. Skaters in the current study reported an average 91 g/day of sugar. While sugary foods may be low in micronutrients, for athletes who need calorie-dense sources of energy, such intakes should not be discouraged [15]. High-sugar, high-fat foods are often the most efficient way to achieve the high-energy diet required to meet the dual energy demands of intense training and growth [15]. Nutrition education efforts should focus on informing athletes and training staff on the macronutrient guidelines for athletes. Current guidelines recommend that athletes, with reference to body weight, should consume 6–10 g/kg carbohydrate and 1.2-1.7 g/kg protein [10]. Intakes below these levels, or intakes that restrict one or more macronutrient, place athletes at risk of micronutrient deficiencies [10]. Particular attention should be paid to the intake of bone-building nutrients like calcium, phosphorus and vitamin D, as female athletes with low energy intakes are at risk for low bone-mineral density [10]. Current guidelines recommend 1000–1500 mg calcium and 400–800 IU Vitamin D for athletes [10]; adequate intake in addition to weight-bearing activity like figure skating may help promote bone strength in these young skaters [34].

The mean EAT-40 score was below the cut-off score of 30 that indicates risk of disordered eating attitudes, and it was comparable to scores of control subjects in the EAT-40 validation study [24]. Ziegler et al. [35] reported a similar mean EAT-40 score of 14.4 in a study of elite skaters; higher EAT-40 scores in that study were associated with lower intakes of micronutrients but not with energy intake. In the current study, elevated EAT-40 scores were associated with older age and BMI but not with reported energy intake. Age and BMI are reported correlates of eating disorder risk among female skaters, as physical changes related to puberty may cause negative self-perceptions [6,29]. Even though the mean EAT-40 score of this young group of skaters was not elevated, they did agree with many items related to restrained eating and preoccupation with weight and food and one-quarter of the skaters had elevated scores. In comparison, the lifetime prevalence of anorexia nervosa and bulimia nervosa in a nationally representative sample of US adolescent females was only 0.3% and 1.3%, respectively [36]. Therefore, skaters need anticipatory guidance to avoid unhealthy weight control behaviors and they should be monitored for signs of caloric restriction or pathogenic weight control. Research suggests nutrition education should consider more than BMI when assessing for energy restriction [16]. Instead, athletes should be encouraged to discuss their body image and body weight concerns to enhance understanding of their dietary practices and satisfaction with current weight and body composition [6]. Training staff should encourage the development of realistic weight and body composition goals and should monitor their own comments or views on appearance to prevent the development of negative self-perceptions among young skaters [6,10].

Limitations of the present study include the reliance on self-reported data and the use of three-day food records. Food and activity records were reviewed with a study staff member, however the collection and review of data were separated by two months. Records may have contained missing or incomplete records that led to misrepresentation of dietary intake and physical activity level. Future studies may combine written instructions with in-person education on the completion of dietary and physical activity records to maximize accuracy. In addition, they may consider shortening the span between collection and review of records, perhaps even utilizing daily review of records to minimize missing or misreported data. Finally, data were collected during training season; the findings of this study may not be generalized to off-season.

## Conclusion

Elite adolescent skaters participate in a lean-build sport where thin, lithe figures are desired. The majority of the sample showed little evidence of eating or weight problems. However, most skaters reported implausibly low energy intakes and one-quarter reported disordered attitudes and behaviors towards eating and body weight control. Skaters should be encouraged to keep their energy intakes in line with the high energy demands of the sport to ensure that their diets are adequate in the nutrients they need for growth, development and training.

## Abbreviations

BMI: Body Mass Index; CDC: Centers for Disease Control and Prevention; DRI: Dietary Reference Intake; EAT-40: Eating Attitudes Test; EER: Estimated Energy Requirement; EI: Energy Intake; NHANES: National Health and Nutrition Examination Survey; USFSA: United States Figure Skating Association.

## Competing interests

The authors declare that they have no competing interests.

## Authors’ contributions

JD, AE and KP drafted and revised the manuscript. WS performed the statistical analysis. KS helped draft the manuscript. PZ conceived of the study and participated in its design and data collection. All authors read and approved the final manuscript.
